# Hydroxytyrosol’s Protective Effect Through Podocalyxin and Pentraxin-3 in Kidney Damage Resulting From Corn Syrup Administration

**DOI:** 10.7759/cureus.73889

**Published:** 2024-11-18

**Authors:** Nevin Kocaman, Elif Onat, Serhat Hançer

**Affiliations:** 1 Department of Histology and Embryology, Firat University Faculty of Medicine, Elâzığ, TUR; 2 Department of Medical Pharmacology, Adıyaman University Faculty of Medicine, Adıyaman, TUR

**Keywords:** corn syrup, hydroxytyrosol, kidney, pentraxin-3, podocalyxin

## Abstract

Introduction: In this study, we aimed to investigate whether hydroxytyrosol (HT) has a protective effect on corn syrup-induced kidney damage in rats and the role of podocalyxin (PCX) and pentraxin-3 (PTX3) in this possible effect.

Methods: Rats were divided into four groups with six rats in each group: 1) control, 2) HT, 3) corn syrup, and 4) corn syrup + HT. Rats were given 30% corn syrup added to their drinking water for six weeks. HT was given orally at 4 ml/kg/day, alone and together with corn syrup. PCX and PTX3 in the renal tissue were assessed through histopathological examination. Biochemical parameters were also examined in the sera with the ELISA method.

Results: In this study, it was observed that PCX and PTX3 levels, which increased as a result of corn syrup administration, decreased after HT treatment (p < 0.001). The increase in amylase, lipase, and insulin levels because of corn syrup consumption decreased with HT consumption (p = 0.001, p < 0.001, p = 0.003, respectively). It was determined that the increase in erythrocyte extravasation, exudate accumulation, and fibrosis observed in the kidney tissue with corn syrup application decreased as a result of HT application (p < 0.001).

Conclusion: It is thought that HT has a protective effect against kidney damage caused by corn syrup and that PCX and PTX3 may play a role in this effect.

## Introduction

Consumption of high fructose corn syrup has increased noticeably in recent years. This situation parallels the further increase in metabolic diseases such as excess weight, non-alcoholic fatty liver disease, cardiovascular disease, and diabetes [1]. Considering the relationship between high blood pressure and diabetes, fructose consumption is thought to be significantly effective in the development of chronic kidney disease (CKD) [2]. In a cross-sectional analysis study, it was observed that the consumption of two or more sugar-containing beverages per day increased the risk of albuminuria [2]. Many studies have shown that fructose consumption increases the likelihood of kidney stone formation [3]. Therefore, it is thought that there is a relationship between excessive fructose consumption and kidney diseases.

Extra virgin olive oil (EVOO) is one of the leading fat and antioxidant ingredients in the Mediterranean diet [4]. The protective effect of EVOO on the kidneys due to its antioxidant properties has been confirmed in experimental nephropathy models. Hydroxytyrosol (HT), a phenolic compound of EVOO, has been shown to have various anti-inflammatory, anti-proliferative, and anti-microbial properties [5]. It has been determined that it has significant effects on preventing the development of cancer, diabetes, CKD, metabolic syndrome, obesity, arterial hypertension, and neurological and cardiovascular diseases [6]. Although the curative effects of HT have been observed in various diseases, the possible underlying mechanisms are still not fully understood.

Podocalyxin (PCX) is a transmembrane O-glycosylated and sialylated protein that is seen especially in podocytes but also exists in neurons, vascular endothelium, and hematopoietic cells [7]. It is located on the apical side of the podocytes and has a network structure that supports the capillaries of the glomerulus. However, they can be seen between cells and in the spaces between podocytes [8]. Podocytes can be damaged by a wide variety of diseases, and as a result, PCX is excreted in the urine. The amount of PCX in urine can provide information about the severity of damage to glomerular epithelial cells [9]. Recently, urinary PCX has begun to be used as a measure of the amount of glomerular destruction and as an indicator of disease progression [10].

Pentraxin-3 (PTX3), the first discovered long pentraxin, is released mainly by monocytes, macrophages, and myeloid dendritic cells stimulated through proinflammatory cytokines such as IL-1β, TNF-α, and Toll-like receptor. Additionally, some amount of PTX3 is released from neutrophils, lymphocytes and endothelial cells [11]. PTX3 effectively participates in the humoral immune response, inflammatory and anti-inflammatory response, and tissue damage and repair [12]. In a wide variety of studies, it has been reported that PTX3 levels are proportional to the severity of sepsis, acute pancreatitis, acute myocardial injury and many diseases. This shows that PTX3 may be a new biomarker to indicate inflammation, infection and tissue damage [13]. Additionally, PTX3 released from neutrophils may be associated with the production of reactive oxygen species (ROS) and vascular endothelial dysfunction [14]. In chronic kidney patients, high PTX3 amount has been shown to be associated with decreased kidney function [15] and this is thought to have a prognostic value in terms of mortality [16].

It is thought that HT has a healing effect against kidney damage caused by corn syrup in rats. Therefore, this study aimed to investigate this protective effect of HT and the role of molecules such as PCX and PTX3 in this possible effect.

## Materials and methods

Animals and experimental design

The study was approved by the Animal Ethics Committee of Adıyaman University (Protocol no: 2024/004). The investigation was conducted according to the "Guide for the Care and Use of Laboratory Animals."

Twenty-four male Sprague-Dawley rats, weighing between 200 and 250 g, aged 8-10 weeks, produced at the Adıyaman University Experimental Research Center, were used in the study. The rats were fed with standard chow and water unlimitedly throughout the study in a fixed environment. Rats were divided into four groups with six animals in each group: 1) control, 2) HT, 3) corn syrup, and 4) corn syrup + HT. No treatment was applied to the control group throughout the study. Liquid HT was supplied by Kale Naturel Herbal Products Company in Turkey. HT was given orally to the animals in groups II and IV at a dose of 4 ml/kg/day for six weeks [17]. Rats in groups III and IV received 30% corn syrup added to their drinking water for six weeks [18]. At the end of six weeks, rats were anesthetized with intraperitoneal ketamine (75 mg/kg) + xylazine (10 mg/kg), and blood was taken from the hearts of rats in all groups. Kidney tissues were placed in 10% formaldehyde solution for histopathological examination.

Serological analyses

Cardiac blood samples of the nonfasted rats were centrifuged at 4℃ and 10.000 g for 30 min. Serum samples were immediately stored at −80℃ until the samples were assayed. Glucose, amylase, lipase, insulin, and uric acid levels were determined by standard enzymatic techniques.

Histochemical examination

Renal tissues of animals were passed through routine histological follow-up series and embedded in paraffin blocks. Hematoxylin and eosin, Masson trichrome, and immunohistochemical stains were applied by taking 5 µm thick sections from these blocks.

Immunohistochemical examination

Standard histological follow-up series were applied to kidney tissue samples of rats and then embedded in paraffin blocks. About 5 µm thick sections were taken from these blocks, and an immunohistochemical staining protocol was performed (19).

In immunohistochemistry (IHC), histological tissue microarray slides of 3 μm thickness were used. The antibodies used were PCX monoclonal antibody (XH358726; Thermo Fisher Scientific, Rockford, USA) and PTX3 Antibody (PA5-36156, Thermo Fisher Scientific, Invitrogen, Waltham, USA). The Zeiss Axio Scope A1 microscope (Carl Zeiss Microscopy GmbH 07745 Jena, Germany) was used for evaluation and documentation. After immunohistochemical staining, a histoscore was created for PCX and PTX3.

As a result of the microscopic evaluation of staining intensity, negative colored areas were given a value of 0, areas showing less than 25% staining were given a value of 0.1, areas showing 26-50% staining were given a value of 0.4, areas showing 51-75% staining were given a value of 0.6, and areas showing staining close to homogeneity (76-100%) were given a value of 0.9. The following formula was used for histoscore [19]:

\[ \text{Histoscore} = \text{Distribution} \times \text{Intensity} \]

Statistical analysis

IBM SPSS Statistics for Windows, Version 22 (Released 2013; IBM Corp., Armonk, New York, United States) was used for statistical analyses. A one-way ANOVA test was performed. The Tukey Honestly Significant Difference (HSD) test was applied for post-hoc multiple comparisons. Results are given as mean ± SD with a significance level of p < 0.05 indicating statistical significance.

## Results

Biochemical findings

As seen in Table 1, plasma amylase, lipase, and insulin were significantly increased in corn syrup-administered rats compared to the control and HT groups (p < 0.001). This situation may be related to the negative effect of corn syrup on glucose metabolism. Plasma glucose, amylase, lipase, and insulin values ​​were lower in the corn syrup + HT group than in the corn syrup group (p = 0.042, p = 0.001, p < 0.001, p = 0.003, respectively). The lowering effect of HT on these values ​​indicates its protective effect on glucose metabolism. Plasma glucose and uric acid values ​​were higher in corn syrup-administered rats compared to the control and HT groups but were not significant. Plasma uric acid values ​​were lower in the corn syrup + HT group than in the corn syrup group but were not significant.

**Table 1:** Levels of various biochemical parameters in blood serum of rats Error bars show SD ^a^p < 0.05 compared to control; ^b^p < 0.05 compared to HT; ^c^p < 0.05 compared to corn syrup HT: hydroxytyrosol

Groups	Control	HT	Corn syrup	Corn syrup + HT
Glucose (mg/dl)	176.67 ± 73.25	200.67 ± 97.58	279.33 ± 130.76	114.33 ± 35.83^c^
Amylase (U/L)	1781.5 ± 44.84	1864.8 ± 97.25	2574.2 ± 284.95^a,b^	1983.3 ± 298.5^c^
Lipase (U/L)	10.33 ± 0.52	11.67 ± 0.82	15.67 ± 1.63^a,b^	10.8 ± 1.33^c^
Insulin (uIU/ml)	0.025 ± 0.16	0.028 ± 0.01	0.07 ± 0.01^a,b^	0.03 ± 0.01^c^
Uric acid (mg/dl)	1.1 ± 0.41	1.57 ± 0.81	4.05 ± 3.53	1.52 ± 0.4

Histochemical findings

**Figure 1:**
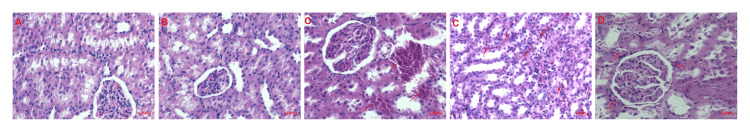
The histopathological examination of renal tissues of observation (hematoxylin and eosin) A) control; B) HT; C) corn syrup; D) corn syrup + HT (arrows indicate changes in tissue) HT: hydroxytyrosol

**Figure 2:**
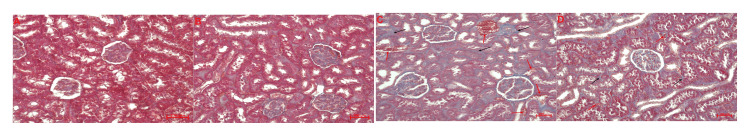
The histological findings of renal tissues of observation (Masson trichrome staining) A) control; B) HT; C) corn syrup; D) corn syrup + HT (arrows indicate changes in tissue) HT: hydroxytyrosol

As a result of the examination of hematoxylin-eosin and Masson trichrome-stained preparations of all groups under a light microscope, the control and HT groups had normal appearances (Table 2, Figures 1-2).

**Table 2:** Histopathologic findings of the renal tissues (hematoxylin and eosin-Masson trichrome) Error bars show SD ^a^p < 0.05 compared to control; ^b^p < 0.05 compared to HT; ^c^p < 0.05 compared to corn syrup HT: hydroxytyrosol

Parameters	Control	HT	Corn syrup	Corn syrup + HT
Erythrocyte extravasation	2.29 ± 0.49	2.43 ± 0.53	6.71 ± 0.49^a,b^	4 ± 0.82^a,b,c^
Exudate accumulation	1.29 ± 0.49	1.14 ± 0.38	6.57 ± 0.53^a,b^	2.43 ± 0.53^a,b,c^
Fibrosis	1.86 ± 0.38	1.71 ± 0.49	6.86 ± 0.38^a,b^	4.29 ± 0.49^a,b,c^

Increased erythrocyte extravasation, exudate accumulation, and fibrosis were observed in the corn syrup group compared to the control and HT groups (p < 0.001). This situation shows the damage caused by corn syrup on the kidney. Compared to the corn syrup group, there was a significant decrease in erythrocyte extravasation, exudate accumulation, and fibrosis in the corn syrup + HT group (p < 0.001). This finding shows the protective effect of HT on the kidney (Table 2, Figures 1-2).

Immunohistochemical findings

**Figure 3:**
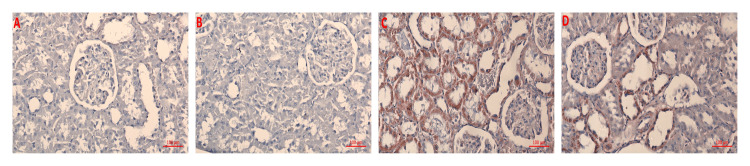
Immunohistochemical results for PCX in renal tissues A) control; B) HT; C) corn syrup; D) corn syrup + HT HT: hydroxytyrosol; PCX: podocalyxin

As a result of the examination of kidney tissue under a light microscope with immunohistochemical staining for PCX and PTX3 immunoreactivity, PCX immunoreactivity increased in the corn syrup group compared to the control and HT groups (p < 0.001). This finding indicates that glomerular damage may occur due to corn syrup consumption. In contrast, PCX immunoreactivity was lower in the corn syrup + HT group than in the corn syrup group (p < 0.001). In this case, HT treatment may have had a protective effect on the damage caused by corn syrup in the glomeruli (Table 3). Histocores showing PCX immunoreactivity for each group are shown in Figure 3.

**Table 3:** Immunohistochemical results for PCX in the renal tissues Error bars show SD ^a^p < 0.05 compared to control; ^b^p < 0.05 compared to HT; ^c^p < 0.05 compared to corn syrup HT: hydroxytyrosol; PCX: podocalyxin

Groups	Control	HT	Corn syrup	Corn syrup + HT
PCX	0.36 ± 0.08	0.34 ± 0.07	0.73 ± 0.16^a,b^	0.41 ± 0.07^c^

**Figure 4:**
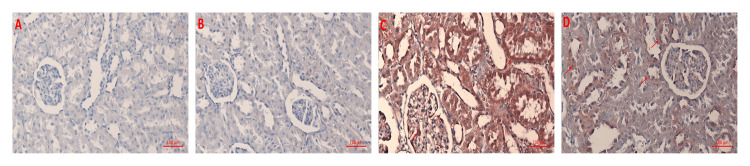
Immunohistochemical results for PTX3 in renal tissues A) control; B) HT; C) corn syrup; D) corn syrup + HT HT: hydroxytyrosol; PCX: podocalyxin

PTX3 immunoreactivity showed a significant increase in the corn syrup group compared to both the control and HT groups (p < 0.001). This finding indicates that corn syrup negatively affects kidney functions. On the contrary, PTX3 immunoreactivity was found to be lower in the corn syrup + HT group compared to the corn syrup group (p < 0.001). In this case, HT has a protective effect on renal functions (Table 4). Histoscores showing PTX3 immunoreactivity for the four groups are shown in Figure 4.

**Table 4:** Immunohistochemical results for PTX3 in renal tissues Error bars show SD ^a^p < 0.05 compared to control; ^b^p < 0.05 compared to HT; ^c^p < 0.05 compared to corn syrup HT: hydroxytyrosol; PCX: podocalyxin

Groups	Control	HT	Corn syrup	Corn syrup + HT
PTX3	0.21 ± 0.07	0.2 ± 0.06	0.69 ± 0.21^a,b^	0.32 ± 0.13^c^

## Discussion

Due to the increase in fructose consumption and the resulting metabolic diseases, research in recent years has focused on the pathophysiological effects of fructose, especially on organs. These studies have increased knowledge about the interaction between fructose, related organ dysfunctions, and diseases. Although various molecular and biochemical mechanisms and their effects and pathophysiology have been elucidated in animal studies, more clinical studies are needed to prevent and treat various diseases that occur due to fructose consumption [1]. In this study, it was tried to investigate whether HT has a protective effect against pathological damage in the kidneys of rats that received corn syrup and the effect of PCX and PTX3 molecules in this. As a result of the study, it was thought that HT has a protective effect against pathological damage in the kidneys caused by corn syrup and that PCX and PTX3 may also play a role in this effect.

The most important of the polyphenolic compounds in EVOO and the main component responsible for the antioxidant effect is HT [20]. When the structure, properties, metabolism, and transformations of the HT molecule were examined, it was seen that its metabolites were mainly excreted through the kidneys [21]. HT accumulates in the kidneys until it is excreted, where it may play a nephroprotective role due to its antioxidant properties [22]. This antioxidant property is important to explain the morphological and functional kidney-protective effect of HT. Furthermore, it has been shown that the use of EVOO with high polyphenol levels in patients with CKD improves the renal profile to a greater extent than the use of EVOO with low polyphenol levels [23]. In our study, consistent with these findings, plasma glucose, amylase, lipase, insulin, and uric acid levels increased with corn syrup application and decreased as a result of HT application, proving once again the protective effect of HT on blood glucose-insulin regulation and the kidney. In addition, it was observed that the increase in erythrocyte extravasation, exudate accumulation, and fibrosis observed in the renal tissue as a result of corn syrup application was significantly reduced as a result of HT application.

PCX is a transmembrane protein located on the apical side of podocytes. It is responsible for regulating podocyte structure and glomerular permeability [24]. Additionally, PCX has been shown to be present in vascular endothelial cells, megakaryocytes, platelets, mesothelial cells, hematopoietic cells, and some neuronal cells. In epithelial cells, PCX plays an important role in many physiological processes, including embryonic development, inflammatory responses, and cancer metastasis, by interacting with adapter proteins involved in actin binding and protein signaling [25]. Although the diagnostic significance of PCX for CKDs is not fully known, early detection of PCX in urine continues to be investigated. These studies revealed that urinary PCX levels are increased in diabetics; in this case, PCX may be a more accurate and precise biomarker in the early diagnosis of diabetic nephropathy compared to albuminuria [26]. Additionally, the level of urinary PCX and the level of urinary podocytes have been found to be associated with the possibility of segmental sclerosis in various glomerular disorders [9]. A relationship has been found between urine PCX and values indicating kidney function (serum creatinine, eGFR, and albuminuria) [27]. Therefore, the amount of PCX in urine may be a biomarker of podocyte dysfunction, indicating the filtration power of the kidney [28]. Consistent with this information in our study, the increase in the amount of PCX in the kidney tissue of rats given corn syrup shows once again that PCX may be an important biomarker indicating kidney damage. Additionally, the decrease in PCX in the HT treatment group supports that HT has a protective effect on kidney damage. Therefore, PCX may be an important target in the diagnosis and treatment of renal pathologies.

PTX3 is a long pentraxin that was first discovered in kidney tissue [29]. PTX3 levels have been observed to increase in acute and CKDs. It has been observed that PTX3 expression increases in renal tubular epithelial cells in ischemia-reperfusion injury of the kidney, as well as in glomerular endothelial cells of patients with IgA nephropathy and type I membranous proliferative glomerulonephritis [30]. Therefore, PTX3 is thought to be effective in the pathogenic process as a pro-inflammatory cytokine [31]. Recent studies have determined that PTX3 does not cause a negative effect on kidney damage [32], and, on the contrary, high PTX3 levels are an important marker of the immune inflammatory response and anti-inflammatory defense mechanism, which probably has a positive effect on the kidneys. Previous studies have shown that PTX3 reduces inflammation, regenerates damaged tissue, and stimulates and protects the immune system [33]. PTX3 binds P-selectin, which enables neutrophils to roll across the membrane of microvascular endothelial cells, thereby reducing leukocyte adhesion and cross-endothelial migration. This will reduce the accumulation of neutrophils and macrophages in the ischemic kidney and stimulate tubular regeneration [32]. It was found that in PTX3-deficient mice, acute ischemic damage of the kidney was worse, and these improved after PTX3 injection [34]. In a different study, it was shown that administration of PTX3 for therapeutic purposes reduced interstitial fibrosis due to acute kidney injury, decreased creatinine levels in serum, and decreased collagen and smooth muscle actin expression. Additionally, it has been observed that the PTX3 level in the kidney tissue is associated with kidney damage in diabetic nephropathy, and PTX3 expression in the kidney is significantly reduced in diabetic nephropathy [35]. In our study, the increase in the amount of PTX3 in the kidney tissue of rats given corn syrup supports the idea that PTX3 increases to create an anti-inflammatory effect in kidney damage. As a result of the information obtained, PTX-3 can be used to measure the extent of kidney damage, early evaluation of renal tubule damage, and monitor its clinical progression. Additionally, better recommendations for clinical treatment can be provided to prevent the development and progression of kidney damage.

Limitations

The most important limitations of the study are that although this study shows that PCX and PTX-3 may be important parameters in the diagnosis and treatment follow-up of kidney damage due to corn syrup consumption, these results need to be supported by different research techniques (western blot, PCR) and confirmed by clinical studies. Since it has a limited sample size, a study with a larger sample size is needed in the future.

## Conclusions

As a result, new biomarkers such as PCX and PTX-3 may be indicators of kidney damage. It was concluded that HT has a protective effect against corn syrup-induced kidney damage, and PCX and PTX-3 may also play a role in this mechanism of action. While HT can be used for the treatment of kidney pathologies, PCX and PTX-3 may also be important in the diagnosis and treatment follow-up of this pathology.
